# A Systematic Review of the Impact of Wildfires on Sleep Disturbances

**DOI:** 10.3390/ijerph181910152

**Published:** 2021-09-27

**Authors:** Fadia Isaac, Samia R. Toukhsati, Mirella Di Benedetto, Gerard A. Kennedy

**Affiliations:** 1 School of Science, Psychology and Sport, Federation University, Ballarat, VIC 3350, Australia; s.toukhsati@federation.edu.au (S.R.T.); g.kennedy@federation.edu.au (G.A.K.); 2 Australian Centre for Heart Health, North Melbourne, VIC 3051, Australia; mirelladb25@gmail.com; 3 School of Health and Biomedical Sciences, RMIT University, Melbourne, VIC 3083, Australia; 4 Institute for Breathing and Sleep, Austin Health, Heidelberg, Melbourne, VIC 3084, Australia

**Keywords:** bushfires, sleep disturbances, trauma, psychopathology, bushfire survivors

## Abstract

Wildfires present a serious risk to humans as well as to the environment. Wildfires cause loss of lives, economic losses, expose people to personal as well as collective trauma, and compromise the mental health of survivors. Sleep disturbances are highly prevalent following a traumatic event; however, their prevalence is not well established amongst those confronted by natural disasters such as wildfires. The aim of this systematic review is to synthesise the empirical findings pertaining to wildfires and the prevalence of sleep disturbances in the general community affected by this natural disaster. We searched EBSCO, PsychINFO, Medline, SpringerLink, CINAHL Complete, EMBASE, PubMed, Scopus and Cochrane Library between January 2012 and March 2021. Five studies met the inclusion criteria. Findings from this systematic review suggest that sleep disturbances, assessed one to ten months following the fires, are highly prevalent in wildfire survivors, with insomnia (ranging between 63–72.5%) and nightmares (ranging between 33.3–46.5%), being the most prevalent sleep disturbances reported in this cohort. Results also highlight the significant associations between sleep disturbances and post-traumatic symptoms following the trauma of wildfires. There is a possible link between sleep disturbance prevalence, severity of, and proximity to fires.

## 1. Introduction

Climate change is posing serious threats to humans and the environment and may be increasing the frequency and intensity of droughts, floods, tornadoes, hurricanes, wildfires, and other extreme weather events. Such weather-related events cause human fatalities, loss of property, massive disruption to infrastructure, economic losses, displacement of those impacted, and negative physical and mental health sequalae [1,2,3].

Wildfires are natural phenomena that deleteriously affect most continents around the world including: Australia, Europe, Asia, and North and South America [4,5,6,7]. In Australia, approximately 20 million hectares were burnt and more than 3000 homes were destroyed in the 2019 summer fires [8]. In the USA, wildfires pose a similar risk to the economy with an annual average loss of $2677 million (USD) [9]. Wildfires also result in injury and the loss of many human lives. Data extracted from the Emergency Event Database shows that fires contributed to the loss of 3753 lives between the year 1901 and 2014, and a further six million peoples’ lives were negatively affected between 1984 and 2013 globally as a result of fires [9].

In addition to injury, loss of lives and economic losses, trauma resulting from wildfires causes disruption to community cohesion and people’s sense of belonging, safety, and wellbeing [10]. Collective trauma takes place when a traumatic event damages the ties that bind community members together and shatters the social fabric of society [11,12]. Hirschberger refers to collective trauma as a loss of identity, affirming that the collective memory persists beyond the single generation within in which it occurs and is remembered by those who are far removed from the traumatic events in space and time [11].

Consequently, wildfires result in increased prevalence of mental health disorders such as depression, anxiety, post-traumatic stress disorder (PTSD), and sleep disturbances [1,8,13]. One of the most noted mental health conditions in the literature following trauma is PTSD. PTSD occurs in people who experience and/or witness, either directly or indirectly (i.e., vicariously), traumatic events such as accidents, natural disasters and personal assaults [14]. In the DSM-5, sleep disturbances including recurrent trauma-related nightmares and difficulties falling or staying asleep are core features of PTSD, and their presentation is a prerequisite for a clinical diagnosis of PTSD [14,15]. While some individuals may experience sleep disturbances following a traumatic experience, not everyone will develop PTSD.

Insomnia is a sleep disturbance present in 30% of the adult population, and is defined as difficulties in initiating and maintaining sleep, frequent nocturnal awakenings and/or suffering from nonrestorative sleep [16,17]. Notably, sleep disturbances were found to be the most prevalent symptoms amongst those surviving other traumatic events such as the earthquake in Japan in 1995 and the Jewish Holocaust [18,19]. Not only do people exposed to disasters show high rates of sleep disturbances, but also frontline and emergency workers who provide support and assistance to survivors. For example, in a sample of 9810 Korean firefighters, Jang et al. found that 50.9% of participants had insomnia [20]. These results suggest that sleep disturbances are more prevalent in those who are repeatedly confronted with trauma than the rates observed in the general population. If left untreated, sleep disturbances can become difficult to treat.

Sleep disturbances can lead to poor physical and psychological health, poor quality of life, and impaired social relations. Further consequences include daytime sleepiness and fatigue, hypertension, diabetes, heart disease, dementia, stroke, migraines, and impaired cognitive functioning including poor concentration and memory, and suicidal ideation [21,22,23,24,25].

Despite the prevalence and negative consequences of sleep disturbances, the literature exploring the impact of wildfires on mental health has focused on mental health outcomes such as substance use, depression, anxiety, and PTSD [26,27,28]. Sleep has been overlooked despite evidence showing that persistent sleep disturbances are a risk factor for the development of psychopathology following trauma [29].

Furthermore, the terminology used in the literature describing sleep disturbances varies with terms such as sleep loss, sleep disturbances, and sleep deprivation all used to describe insomnia [29]. This creates a host of problems such as, lack of clarity in defining sleep disturbances and confusion about precision in relation to sleep disturbance estimates. 

The aim of this review was to synthesise the literature and explore the prevalence of sleep disturbances in wildfire survivors in the general public. In doing so, this review provides information that is essential for appropriate planning for health care needs [30]. Furthermore, detecting the prevalence of health conditions/sleep is important in revealing potential causes of the condition and uncovering the burden in relation to life expectancy, quality of life, morbidity, and other factors [31]. Learning more about sleep disturbances in those exposed to traumatic events such as wildfires may inform policy makers navigating where investments in health care should be targeted, such as the provision and timing of treatments [32]. Subsequently, this may reduce both the burden of sleep disturbances and the subsequent development of serious psychopathology in communities [29,33].

## 2. Method

### 2.1. Protocol and Registration

Utilising the Preferred Reporting Items for Systematic Reviews and Meta-Analyses (PRISMA, Berlin, Germany) we conducted a systematic review to synthesise empirical findings on the topic of wildfires and the prevalence of sleep disturbances in the general community [34]. A protocol for this systematic review was registered on PROSPERO on the 17 February 2021, CRD42021231659 [35].

### 2.2. Search Strategy

Our search strategy was formulated following the PICO principle (population, intervention, comparison, outcome) [36]. We searched EBSCO, PsychINFO, Medline, SpringerLink, CINAHL Complete, EMBASE, PubMed, Scopus and Cochrane Library between January 2021 and March 2021. In addition, Google Scholar and the reference lists of publications were also utilised. Table 1 shows the combination of search terms that were used to search the various databases. Search terms were specified prior to starting the search. The same search terms were used across all databases to optimise findings, with the exception of Scopus and SpringerLink databases (for which no results were obtained using the pre-determined search terms) and as such, search terms were modified but retained key terms (such as wildfires and sleep) across all search variations. 

### 2.3. Inclusion Criteria

Literature published in English between January 1990 and March 2021 exploring the impact of wildfires on sleep was included. We decided to include children as well as adults in this systematic review. For our final analysis we only considered peer-reviewed articles.

### 2.4. Exclusion Criteria

Excluded studies were: (1) statements; (2) commentaries; (3) studies unrelated to wildfires; (4) studies excluding sleep disturbances; (5) animal studies; (6) published prior to 1990; (7) not peer reviewed; (8) concerned with firefighters or emergency workers; and (9) studies published in a language other than English. 

### 2.5. Study Selection

Initial assessment and screening of the title and abstract were performed by one reviewer (FI), and those deemed suitable, were then screened for a full text assessment. Reported data and background information for each study were extracted and summarised consistent with the PICO method. Upon the selection of final articles, data were checked by a second reviewer (GK). There was a 100% inter-rater agreement between the reviewers (FI and GK).

### 2.6. Quality Assessment

A risk of bias was performed utilising the Joanna Briggs Institute Critical Appraisal Checklist for studies reporting prevalence data (JBI) [37].

## 3. Results

**Figure 1:**
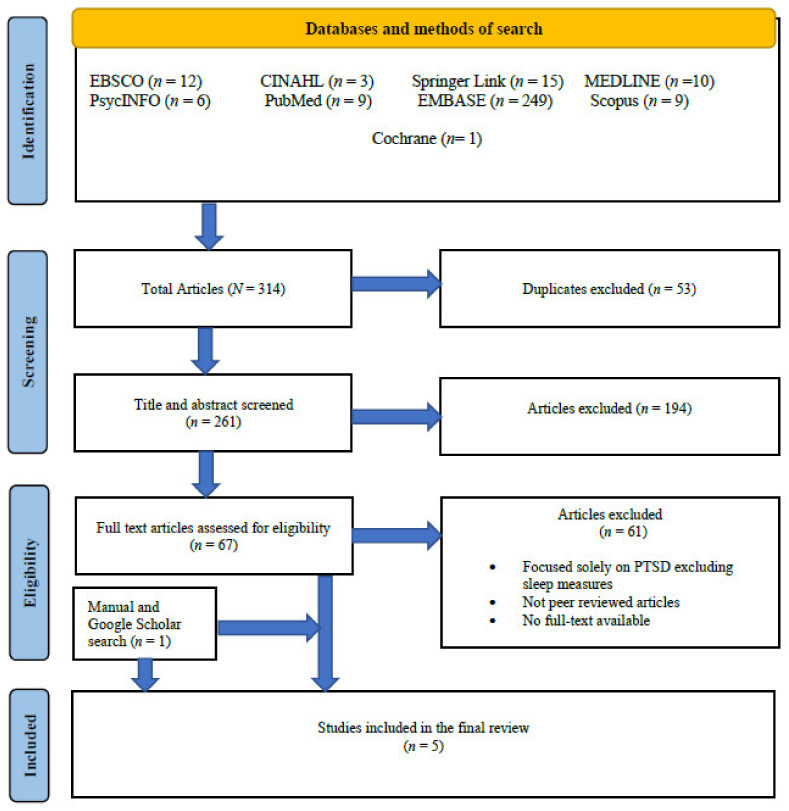
PRISMA flow diagram of the databases search and selection of final studies.

A total of 314 studies were identified for screening by title and inclusion criteria. Following the exclusion of 53 duplicates, a total of 194 studies were further screened by title and abstract and were excluded for not meeting the inclusion criteria. Most of these articles did not address the prevalence of sleep disturbances, were not peer reviewed, fires were not due to natural disasters, and the study sample did not include the general public. Following this, 67 studies were screened by abstract and full article, of which, a further 61 were excluded leaving six studies. One additional study was located by checking reference lists and Google Scholar search. A total of seven studies met the inclusion criteria. However, two studies were excluded following correspondence with the authors in May 2021 [38], due to data overlap in two different papers [39]. Five studies met the inclusion criteria, for which there was 100% inter-rater agreement (see Figure 1).

Table 2 provides a summary of the selected studies, published between 1990–2021, and includes the country of origin, timeline of sleep disturbance assessments following fires, how sleep disturbances were measured, and prevalence data of sleep disturbances. Further information was provided upon request via email correspondence with Dr Mishra Jyoti on the 12th of April 2021. Table 3 provides a detailed account of the risk of bias appraisal utilising the Joanna Briggs Institute JBI Checklist [37].

### 3.1. Findings from the Included Studies

#### 3.1.1. Prevalence of Sleep Disorders

Two studies provided prevalence data derived from clinical diagnosis of sleep disturbances. Clinical diagnosis entails a health professional undertaking a clinical interview with participants to establish diagnosis of sleep disorders. Following a clinical interview, insomnia rates ranged from approximately 43.6% to 98.7%, followed by presumptive sleep disordered breathing and chronic nightmare disorders [40,42]. Both studies utilised highly reliable and valid measures to diagnose insomnia such as: The Clinician Administered PTSD Scale (CAPS #E6) [45], The Sleep Medicine History [46], and Sleep-Disordered Breathing Diagnostic Criteria [47]. The difference in prevalence between the two studies is perhaps attributable to the design of the two studies. The sample in Krakow et al.’s [42] study targeted participants with sleep complaints seeking treatment for post-traumatic sleep disturbances, whereas Belleville et al.’s study surveyed community members affected by the 2016 fires in Fort McMurray and assessed psychological and sleep disturbances following the fires [40]. Whilst, the sample selected by Krakow et al. [42] is self-identified as being symptomatic hence participants were seeking treatment for their sleep disorders following the fires, the sample in Belleville and colleagues [40] was randomly selected, by research assistants, from the community following the fires. One can argue that both samples are susceptible to selection bias, however the sample in Belleville et al.’s study seem to be more representative of the prevalence of sleep disorders in a community sample following the fires [40]. 

Studies adopting a non-diagnostic approach, sleep disturbances were assessed using self-report scales or questionnaires, reported insomnia prevalence between 63% and 72.5% [40,43]. These two studies utilised highly reliable and valid measures in the assessment of sleep disturbances such as, the Insomnia Severity Index ISI, the Athens Insomnia Scale ASI, The Pittsburgh Sleep Quality Index and its Addendum PSQI-A, and the Pittsburgh Sleep Quality Index PSQI [48,49,50,51,52]. As highlighted in the assessment of risk of bias, all three studies providing prevalence data used valid and reliable measures to assess sleep disturbances (Table 3) [40,42,43].

#### 3.1.2. Prevalence of Sleep Disturbances in Children

One study in this systematic review provided prevalence of sleep disturbances in children. Jones et al. [41] assessed 22 children following the 1990 wildfires in South California. The researchers assessed the sample at six weeks and at ten weeks following the fires, and divided the participants into two group; children with high loss defined as those whose families sustained significant damage or loss to their homes, and children with low loss whose families sustained relatively little loss to their homes. Results revealed that at six weeks assessment, children who experienced high loss had higher rates of recurrent dreams about the fires and symptom of insomnia (46.2% and 69.2%, respectively), than those who were identified as low loss children (33.3% for both recurrent dreams and insomnia). At ten weeks, the high loss group also scored higher on insomnia (84.6%) and on recurrent dreams (53.8%), than children in the low loss group on insomnia (44.4%) and on recurrent dreams (55.6%) [41].

#### 3.1.3. Most Prevalent Sleep Disturbances

In this systematic review, insomnia was found to be the most prevalent sleep disturbance [40,41,42,43], followed by nightmares ranging between 33.3% and 46.5% [42,43]. Only one study provided data on other sleep disorders such as sleep disordered breathing (94.8%) and sleep apnoea (41%) of the sample [42]. 

#### 3.1.4. Prevalence of Sleep Disturbances and PTSD

Four studies identified a significant correlation between sleep disturbances and post-traumatic stress symptoms or PTSD. Insomnia was present in 79.1% of those diagnosed with PTSD; likewise, nightmares were more prevalent in those with PTSD compared to those without PTSD (46.5% versus 12.3%, respectively) *p* = 0.002 [43]. Insomnia, nightmare severity, and impairment of sleep-disordered breathing were significantly correlated with post-traumatic symptoms of hyperarousal and intrusion (*p* < 0.001) [42]. Belleville and colleagues [40] also found that sleep disturbances, sleep quality and insomnia were significantly associated with PTSD three months post-fires (*p* < 0.01). Furthermore, higher levels of sleep disturbances on the Patient-Reported Outcomes Measurement Information System (PROMIS) were associated with higher scores on the PTSD-Checklist (PCL-5, Blevins et al., 2015) [44]. 

#### 3.1.5. Proximity to Fires, Gender, Age and Sleep Disturbances

Overall, studies in this systematic review indicated that sleep disturbances can be reactive to the experience of individuals and their proximity to fires. Sleep quality was significantly worse for those who were directly exposed to fires than for those who were indirectly exposed or who only heard about the fires, *p* < 0.001 [44]. Furthermore, scores on the PTSD-Checklist (PCL-5) [53] were significantly higher for the directly exposed group than the indirectly affected group [44]. Similarly, insomnia was significantly more prevalent in participants who reported being in danger during the fires and those scoring higher on the “fear of imminent death” scale, *p* = 0.005 [43]. Additionally, children who experienced high personal loss experienced more difficulties in falling asleep than those who reported low loss (84.6% and 44.4%, respectively) [41]. This suggests that those who had a more confronting experience with fires, showed more severe symptoms of PTSD and reported worse sleep quality than those that were indirectly affected by the fires. Further to that, one study [43] examined the association of demographic factors and sleep disturbances; it was found that being a female and being older had 3.16 times and 1.04 times greater likelihood for having insomnia. Other studies in this systematic review either did not explore such association or found no association between demographic factors and sleep disturbances. 

#### 3.1.6. Timing of Sleep Disturbance Assessment Relative to Fire Occurrence

The studies reviewed assessed sleep disturbances at different time points following the fires, ranging from one month following the fires [43] to ten months post-fires [42]. The studies are cross sectionally designed as such they provide limited understanding about how sleep changes overtime post-fires. Future longitudinal studies could provide evidence of how prevalence of sleep disturbances change overtime.

## 4. Discussion

The aim of this systematic review was to explore the prevalence of sleep disturbances in the general public affected by wildfires. Only five studies met the inclusion criteria. The review found that there was a wide variation in the prevalence of sleep disturbances amongst wildfire survivors. The prevalence of sleep disturbances for insomnia diagnosed by a clinician was found to be 43.6% in the general public. This rate was higher (i.e., 72.5%) for insomnia in non-clinical samples using self-report measures [40,42,43]. The reported prevalence of 72.5% in this review, in a non-clinical sample, is higher than the rate reported by Jang and colleagues in their sample of firefighters (*N* = 9810, 50.9%) [20], and higher than that reported in the general population of 30% [16,17]. The rate of prevalence in this review is also higher than those reported in other natural disasters. For example, in a community sample of 2593, 14 months following the Japan earthquake and tsunami in 2011, Matsumoto and colleagues found that sleep disturbances were reported by 15% of their sample [54]. The difference between the prevalence rates reported here and those reported by other researchers may be attributed to differences in methodologies used to measure sleep and the definition of sleep disturbances [29,54].

Findings from this systematic review provided prevalence of insomnia and recurrent dream symptoms in children. Results from this study showed an increase in symptoms of insomnia and recurrent dreams about the fires at both six-and ten-weeks assessments following the fires for the high loss children compared to the low loss group [41]. These findings are consistent with those of past research that has reported similar findings in children exposed to natural disasters. Lai et al. [55] examined sleep in 269 children following Hurricane Ike in 2008, at 8 months and 15 months post-disaster. Sleep problems persisted from time 1 at (8 months) to time 2 at (15 months) measurement; with children reporting difficulties in falling asleep (49% at both measurements), difficulties maintaining sleep (42% to 39%) and sleeping more than usual (45% to 43%) [55]. This shows that sleep problems in children persist and may also increase over time following the trauma of a natural disaster [41,55]. 

The prevalence of nightmares ranged between 33.3% and 46.5%, and were found to be the second most prevalent sleep disturbance in wildfire survivors in this review [42,43]. Research exploring other types of traumas also reported high prevalence rates of nightmare in veterans with and without sexual trauma and in adults with PTSD [56,57,58,59,60,61]. It is difficult to compare prevalence rates emerging from this systematic review with findings from other research studies. Research indicates that the prevalence of nightmares and their severity is governed and is likely dictated by the type of trauma individuals experience. For instance, in a study of 4440 children and youth aged between 7–18 years old, Secrist and colleagues assessed the prevalence of nightmares and their relationship with the type of experienced trauma [62]. Nightmares were deemed to be “clinically significant” if they were experienced twice or more per week. The researchers found that 33.1% of this sample reported “clinically significant” nightmares. Moreover, of those who experienced sexual abuse, 21.1% were more likely to experience “clinically significant” nightmares; and of those who experienced medical trauma, 10.2% were least likely to experience “clinically significant” nightmares. Other types of traumas such as community violence, domestic violence, physical abuse, natural disasters, and death trauma showed different associations with nightmares and their severity. Participants were 1.3 times more likely to report experiencing “clinically significant” nightmares for every additional encountered trauma [62]. This suggests that not only the type of trauma that is endured by individuals but also cumulative trauma that someone may experience is likely to increase the prevalence of sleep disturbances. 

The study by Silveria and associates reported that cumulative trauma (i.e., childhood trauma) significantly increased the risk of sleep disturbances and predicted poorer sleep quality in wildfire survivors [44]. Moreover, in their review of studies published in 2018, Lowe and colleagues examined the impact of disasters on PTSD and other mental health conditions following the occurrence of natural disasters [13]. The researchers stated that cumulative trauma can increase the risk of poor outcomes on mental health in a dose–response fashion. Furthermore, the lack of social support and community belonging moderated the relationship between trauma and sleep disorders [13]. Notably, a recent study confirmed the importance of social interaction and how this has changed as a result of COVID-19 compromising mental health conditions such as depression and anxiety [63]. This is reflective of the impact of collective trauma, referred to earlier in this review [11]. This calls for the need to cater for such variables when exploring the association between wildfire trauma and sleep disturbances. 

Findings from this systematic review also highlighted the high prevalence of sleep disturbances in those with post-traumatic stress symptoms and/or PTSD. More specifically, findings derived from this review indicate a higher prevalence of both insomnia and nightmares, 79.1% and 46.5%, respectively, in those with PTSD or post-traumatic stress symptomatology compared to those without PTSD [40,42,43,44]. A wealth of literature supports this significant association and suggests a bi-directionality between the two conditions [29,59,64]. Despite this, leaders in sleep research now affirm that sleep disturbances are stand-alone disorders and they deserve exclusive attention regardless of whether they were initiated by other health conditions [65,66]. The inclusion of sleep disturbances being a hallmark in the diagnosis of PTSD [15] may inflate the reported prevalence of sleep disturbances in this population [29,67]. To illustrate, Breslau and colleagues [67] carried out a 10-year follow up research on a community subsample (*n* = 292). At baseline, participants did not meet PTSD diagnosis. Between 1994 and 1999, 25% of participants who were exposed to trauma developed PTSD. In those diagnosed with PTSD, 87% of the sample reported sleep disturbances (indexed using self-report measures). However, when objective measures of sleep such as polysomnography was implemented, no significant differences were detected between those with and without PTSD in either sleep initiation or sleep maintenance [67]. This is perhaps indicative of the need to consider both objective and self-report measures when assessing sleep disturbances to gain a complete and an accurate presentation of sleep disturbances.

Another factor for consideration when examining sleep disturbances in wildfire survivors is proximity to, and experience with fires. Two studies in this review provided data on the importance of assessing how proximity, in both children and adults, and the experience with fires can impact sleep disturbances with those directly exposed to fires experiencing higher prevalence of sleep disturbances and poorer sleep quality than those non-directly exposed [41,44]. Research on other types of disasters confirm such association. Tempesta et al. [68] assessed sleep quality of 4993 residents, two years following the 2009 L’Aquila earthquake in Italy, utilising subjective sleep quality measures such as the PSQI and PSQI-A. Researchers in this study examined sleep quality of two different subsamples from pre, *n* = 754, to post, *n* = 665, earthquake impact of sleep quality and found a significant decline in sleep quality from pre assessment to post assessment 24 months following the earthquake, *p* < 0.001. They also tested the proximity of other subsamples relative to distance from the epicentre and found that the group, *n* = 739, living within 40 km radius of the disaster showed the highest incidence of disturbed nocturnal behaviour and lowest sleep quality in comparisons to groups living further away *p* < 0.001. Beyond the distance of about 70 km radius, sleep quality scores were found to be within the normal range [68]. Disaster survivors’ research suggests that the loss of loved one, injury and property damage, the loss of internal and external resources are the most important factors in predicting mental health and can predict the recovery of individuals [1,69,70].

One study in this systematic review found a significant association between age, gender and sleep disturbances [43]. Other studies support this finding, being a female and being older is associated with higher rates of sleep disturbances and higher likelihood of prolonged sleep difficulties [54,57,59]. 

In relation to sleep disturbances and time point of assessments, without longitudinal studies it is difficult to reach a conclusion on how sleep disturbances and their frequency change over time. The selected studies measured sleep disturbances at different intervals ranging from one month to ten months following the fires [42,43]. Therefore, the findings from this systematic review are not reflective of how sleep may change overtime following the fires. Other studies carried out on survivors of natural disasters report stability of severity and worsening of symptoms for specific conditions across time in the aftermath of disasters [68,71,72]. Researchers call for continued need to monitor the symptomology of those affected by disasters and the identification of those who are most vulnerable in the aftermath of a natural disaster; which aids in allocating appropriate resources and treatments to ameliorate the risks associated with sleep disturbances [13,72,73].

In interpreting the findings from this systematic review, a number of factors must be considered. Some of the limitations include the small number of studies eligible for inclusion due to the limited research in this area, and the heterogeneity of methodologies and outcome measures which precluded a meta-analysis. Despite these limitations, the findings from this systematic review are novel in the field of wildfires and sleep, and highlight the high prevalence and the severity of sleep disturbances in wildfire survivors. 

## 5. Conclusions

Wildfires pose a serious risk of injury to humans, both directly by causing physical or psychological injury, and/or indirectly by exposing people to the trauma of losing lives, death of relatives, neighbours, financial hardship and breaking the social ties with others [1,10,13]. This systematic review highlighted the high prevalence of sleep disturbances among wildfire survivors, the significant association of sleep disturbances and post-traumatic symptomology, the importance of the need to cater for the type, the magnitude and proximity to trauma and its impact on sleep disturbances. The area of wildfires and sleep disturbances needs further refinement to establish a more comprehensive system for measuring sleep disturbances in wildfire survivors.

## Figures and Tables

**Table 1:** Keywords and databases search.

Database	Keywords
EBSCO	(sleep-wake disorder * OR insomnia OR insomniac OR delayed sleep phase disorder * OR sleep apnea OR sleep apnoea OR parasomnia sleep deprivation OR sleep paralysis OR night sweats OR REM sleep disorder * OR excessive sleep OR sleep walking OR hypersomnia OR circadian rhythm sleep disorder OR narcolepsy OR RLS OR restless leg syndrome OR REM sleep behaviour disorder * OR REM sleep behavior disorder * OR night terrors OR bruxism OR sleep movement disorder * OR sleep related breathing disorder * OR sleep onset OR sleep maintenance OR non-24 h sleep wake disorder OR nightmare OR nightmares) AND (bushfires OR wildfires OR wildland fires OR forest fires OR brushfires)
PsychINFO	
Medline, CINAHL	
Complete	
EMBASE	
PubMed	
Cochrane Library	
Scopus	((bushfires OR wildfires OR wildland fires AND fires OR brushfires) AND (sleep AND disorders OR insomnia OR nightmares))
SpringerLink	Bushfires AND wildfires AND sleep disorders AND sleep difficulties AND PTSD

**Table 2:** Summary of studies published between 1990 and 2021 examining the impact of bushfires on sleep quality in bushfire survivors.

Authors	Country	Period Following the Fires	Sample Size	Measures of Sleep	Summary of Findings
Belleville et al., 2019 [40]	Canada	Three months after the 2016 wildfires in Fort McMurray	379 adult evacuees (subsample of 55 adult completed diagnostic interview)	CAPS #E6 ISI PSQI-A PSQI	60% of the sample had a provisional diagnosis of PTSD. Repeated disturbing memories were reported by 77.4% [95% CI: 72.90–81.35] of their sample, 76.7% [95% CI: 72.13–80.65] reported feeling upset when reminded of the stressful experience and 72.5% [95% CI: 67.78–76.75] reported trouble falling or staying asleep. In a subsample of 55 individuals, 29.1% [95% CI: 18.77–42.14] met the clinical criteria for PTSD with 43.6% [95% CI: 31.38–56.73] of the sample receiving diagnosis of insomnia.
Jones et al. [41]	USA	Six weeks following the 1990 Wildfires Southern California	13 children in the High Loss group HL (M = 9.1 years) 9 children in the Low Loss group LL (M = 9.8 years)	Items derived from the Diagnostic Interview for Children and Adolescents	The measure of PTSD showed the following: item, “dreaming about it repeatedly” (HL 46.2% VS. 33.3%LL). Item, “I had trouble falling asleep/staying asleep” (HL 69.2% VS. 33.3% LL). Impact of Event Scale (IES) was administered a month following the first measurement. The two groups reported the following: item, I “had trouble falling asleep or staying asleep because of a picture or a thought about it that came into my mind” (HL 84.6% VS 44.4% LL). Item, “I had a dream about it” (HL 53.8% vs. 55.6% LL).
Krakow et al. [42]	New Mexico	Six to ten months following the 2000 Cerro Grande Fire	78 adult survivors of the fire	SMH SDBDC Autoset Portable II DDNSI FOSQ global ISI	Most participants, 98.7%, had psychophysiological insomnia, 94.8% of participants had presumptive sleep disordered breathing and 33.3% had chronic nightmare disorder. The insomnia symptoms were in the moderate to severe range. Sleep quality was rated fair to poor by those who suffered from insomnia. 92% of the sample reported morning dry mouth, morning headache. Nocturia was reported by 86% of the sample. Dry mouth upon awakening was reported by 51%. Morning headache was reported by 29%. Obstructive sleep apnoea was reported by 41% and 54% reported predominantly upper airway resistance 31% of the sample presented with classic snoring and obstructive sleep apnoea, and 69% presented with atypical symptom.
Psarros et al. [43]	Greece	One month following the fires of August 2007	92 adult survivors of the fires	AIS	63.0% [95% CI: 53.0–73.1%] of the sample reported symptoms of insomnia, with 57.6% of the sample reported awakenings during the night followed by 34.8% of the sample reporting delayed sleep induction Nightmares were found to be significantly different, *p* = 0.002, between those with PTSD 46.5% and those without PTSD 12.3%.
Silveira et al. [44]	USA	Six months post the 2018 Camp fire California	725 adult residents affected by the fires	PROMIS	Scores on the PCL-5 Post-Traumatic Stress Disorder were significantly higher in directly exposed individuals (*n* = 124 not exposed, *n* = 201 indirectly exposed, *n* = 147 directly exposed) with *B* regression weight reported to be (−3.88, 1.95 and 9.54, respectively) PTSD/PCL-5 scores were positively correlated with childhood trauma. Directly Exposes Sleep Quality (PROMIS) < indirectly Exposed < Nonexposed (*p* < 0.001) Higher sleep disturbance (PROMIS) scores predicted higher PCL-5 scores.

*Note*. AIS = The Athens Insomnia Scale; CAPS: The Clinician Administered PTSD Scale; DDNSI = Disturbing Dream and Nightmare Severity Index; FOSQ global = Functional Outcomes of Sleep Questionnaire; ISI = Insomnia Severity Index; PCL-5 = PTSD-Checklist; PROMIS = Patient-Reported Outcomes Measurement Information System Sleep disturbance scale; PSQI-A = The Pittsburgh Sleep Quality Index and its Addendum for PTSD; PSQI = Pittsburgh Sleep Quality Index; PTSD = Post Traumatic Stress Disorder; SDBDC = Sleep-Disordered Breathing Diagnostic Criteria; SMH = Sleep Medicine History.

**Table 3:** Risk of bias appraisal.

JBI Checklist Appraisal Tool	Included Studies				
	Belleville et al. [40]	Jones et al. [41]	Krakow et al. [42]	Psarros et al. [43]	Silveira et al., 2021 [44]
Was the sample frame appropriate to address the target population?	Yes	Yes	Yes	Yes	Yes
Were study participants sampled in an appropriate way?	Yes	Yes	Yes	Yes	Yes
Was the sample size adequate?	Yes	No	Yes	Yes	Yes
Were the study subjects and the setting described in detail?	Yes	Yes	Yes	Yes	Yes
Was the data analysis conducted with sufficient coverage of the identified sample?	Yes	Yes	Yes	Yes	Yes
Were valid methods used for the identification of the condition?	Yes	No	Yes	Yes	No
Was the condition measured in a standard, reliable way for all participants?	Yes	No	Yes	Unclear	Yes
Was there appropriate statistical analysis?	Yes	Yes	Yes	Yes	Yes
Was the response rate adequate, and if not, was the low response rate managed appropriately?	Yes	Yes	Yes	Yes	Yes
